# Potency of Full- Length MGF to Induce Maximal Activation of the IGF-I R Is Similar to Recombinant Human IGF-I at High Equimolar Concentrations

**DOI:** 10.1371/journal.pone.0150453

**Published:** 2016-03-18

**Authors:** Joseph A. M. J. L. Janssen, Leo J. Hofland, Christian J. Strasburger, Elisabeth S. R. van den Dungen, Mario Thevis

**Affiliations:** 1 Department of Internal Medicine, Division of Endocrinology, Erasmus MC, Rotterdam, the Netherlands; 2 Department of Medicine for Endocrinology, Diabetes and Nutritional Medicine Charité Universitätsmedizin, Berlin, Germany; 3 Center for Preventive Doping Research – Institute of Biochemistry, German Sport University Cologne, Cologne, Germany; Thomas Jefferson University, UNITED STATES

## Abstract

**Aims:**

To compare full-length mechano growth factor (full-length MGF) with human recombinant insulin-like growth factor-I (IGF-I) and human recombinant insulin (HI) in their ability to activate the human IGF-I receptor (IGF-IR), the human insulin receptor (IR-A) and the human insulin receptor-B (IR-B), respectively. In addition, we tested the stimulatory activity of human MGF and its stabilized analog Goldspink-MGF on the IGF-IR.

**Methods:**

The effects of full-length MGF, IGF-I, human mechano growth factor (MGF), Goldspink-MGF and HI were compared using kinase specific receptor activation (KIRA) bioassays specific for IGF-I, IR-A or IR-B, respectively. These assays quantify activity by measuring auto-phosphorylation of the receptor upon ligand binding.

**Results:**

IGF-IR: At high equimolar concentrations maximal IGF-IR stimulating effects generated by full-length MGF were similar to that of IGF-I (89-fold vs. 77-fold, respectively). However, EC50 values of IGF-I and full-length MGF for the IGF-I receptor were 0.86 nmol/L (95% CI 0.69–1.07) and 7.83 nmol/L (95% CI: 4.87–12.58), respectively. No IGF-IR activation was observed by human MGF and Goldspink-MGF, respectively. IR-A/IR-B: At high equimolar concentrations similar maximal IR-A stimulating effects were observed for full -length MGF and HI, but maximal IR-B stimulation achieved by full -length MGF was stronger than that by HI (292-fold vs. 98-fold). EC50 values of HI and full-length MGF for the IR-A were 1.13 nmol/L (95% CI 0.69–1.84) and 73.11 nmol/L (42.87–124.69), respectively; for IR-B these values were 1.28 nmol/L (95% CI 0.64–2.57) and 35.10 nmol/L (95% 17.52–70.33), respectively.

**Conclusions:**

Full-length MGF directly stimulates the IGF-IR. Despite a higher EC50 concentration, at high equimolar concentrations full-length MGF showed a similar maximal potency to activate the IGF-IR as compared to IGF-I. Further research is needed to understand the actions of full-length MGF in vivo and to define the physiological relevance of our in vitro findings.

## Introduction

**Fig 1:**
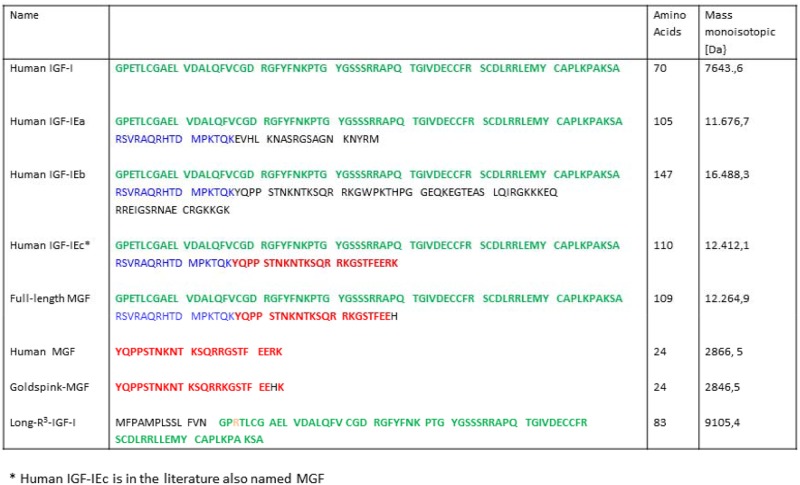
Sequence, number of amino acids and molecular weight of human IGF-I, three human pro-IGF-I isoforms, full-length MGF, Human MGF, Goldspink-MGF and Long R3-IGF-I (modified from Thevis et al. (6)).

Insulin-like growth factor-I (IGF-I) is considered a key regulator of muscle development and growth [1]. For skeletal muscle, IGF-I in conjunction with additional growth factors, coordinates myoblast proliferation, differentiation, and fiber formation during normal growth as well as during regeneration after injury [1]. The IGF-I gene spans more than 90 kb of chromosomal DNA and generates three isoforms. Alternative splicing of IGF-I pre-mRNA can produce three multiple mRNA species, depending on the inclusion of alternative leader and C-terminal exons [2]. All mRNA splice variants contain exons 3 and 4, which encode the mature 70-amino-acid IGF-I peptide consisting of the B, C, A, and D domains. All three mRNA splice variants encode C-terminal extensions termed E-domains to denote their positions relative to the BCAD domains of mature IGF-I: mRNAs containing exon 4 spliced to exon 6 are designated as IGF-IEa, mRNAs containing exon 5 spliced to exon 4 have been designated as IGF-IEb, whereas those containing exon 4 spliced to exon 5 and exon 6 are designated IGF-IEc in humans (Fig 1)[2]. Due to the apparent role of IGF-IEc up-regulation in muscle remodeling, IGF-IEc is also named mechano growth factor (MGF) [2, 3]. Rather than acting in an endocrine fashion, IGF-IEc produced in the body is more likely functioning in a paracrine/autocrine mode [4]. For example, IGF-IEc produced in skeletal muscles in response to mechanical stimuli or damage, functions in skeletal muscles probably as a local muscle growth/repair factor [5]. It has been further suggested that after injury or training locally produced IGF-IEc in muscles is required to activate muscle stem (satellite) cells to divide thereby providing extra nuclei necessary for muscle repair and hypertrophy [5].

A single injection of MGF-cDNA containing plasmid vector (which corresponds to IGF-I Ec in human) resulted in a 25% increase in the mean muscle fiber cross-sectional area of the injected normal muscle within two weeks [4]. Consequently, IGF-IEc represents a potential drug candidate for the treatment of several neuromuscular disorders. However, its potential misuse as doping in sport games has been also identified and therefore the use of IGF-IEc have been prohibited according to the regulations of the World Anti-Doping Agency (WADA) since 2005 [6].

In 2014 a formerly unreported recombinant and non-natural protein was identified and characterized by state-of-the-art analytical approaches [6]. The molecular sequence of this unknown substance is closely related to human IGF-IEc: the C-terminus has been found to be modified compared to human IGF-IEc by replacing the terminal lysine and arginine residues by a histidine (See Fig 1). Therefore this unknown substance has been denoted full-length MGF and can be considered as an analog of human IGF-IEc.

The insulin-IGF system includes at least four receptors: the IGF-I receptor (IGF-IR), the IGF-II receptor, the insulin receptor-A (IR-A) and the insulin receptor-B (IR-B). Both the IGF-IR and the IRs belong to the family of ligand-activated receptor kinases. Binding of a ligand to the extra-cellular alpha subunit of the IGF-IR or the IRs induces the receptors to undergo a conformational change, which enables autophosphorylation of the intrinsic tyrosine kinase domains of the beta-subunits of the IGF-IR and the IRs, respectively. Several lines of evidence indicate that autophosphorylation of the IGF-IR and IRs initiates the majority of the biological effects mediated by IGF-I and insulin.

Kinase Receptor Activation (KIRA) bioassays make use of cell lines stably transfected with receptors and quantify ligand bioactivity by measuring ligand-induced receptor tyrosine kinase activation in terms of receptor-phosphorylation [7]. In our laboratory we have established KIRA bioassays specific for the human IGF-IR, human IR-A and human IR-B, respectively [8, 9]. This allows us to specifically compare the activity of full-length MGF with IGF-I and insulin with respect to their ability to activate the IGF-IR, IR-A and IR-B. In addition, we tested whether stimulating activity of full-length MGF on the IGF-IR, IR-A and IR-B differs in action from human MGF and its stabilized analog Goldspink -MGF [3, 10].

## Materials and Methods

### Materials

The human embryonic kidney cell-line (HEK) Flip-in^™^-293 was obtained from Invitrogen life technologies (Breda, The Netherlands). Plasmids (pNTK-2) containing a cDNA insert of the human IR-B(pNTK2-IR-B) or IR-A (pNTK2-IR-A) were kindly provided by Axel Ullrich (Martinsried, Germany). The HEK IGF-IR cell-line was a kind gift from P. de Meyts (Gentofte, Denmark). Dulbecco's Modified Eagles Medium (DMEM: gluc+, L-Glutamin +,Pyr+), Penicillin/ Streptomycin, Hygromycine, Geneticin, Fugene^®^ transfection reagens and FBS were obtained from Invitrogen life technologies (Breda, The Netherlands). Human Serum Albumin (HSA), (Octalbine^®^) was obtained from Octapharma (Lachen, Switzerland). Culture plates (flat-bottomed 48 wells) were obtained from Corning Costar (Schiphol, The Netherlands). Microtiter 96-wells plates were purchased from Perkin Elmer-life sciences (Groningen, The Netherlands). Antibody coating buffer contained 15mM sodium carbonate and 35mM sodium hydrogen carbonate (pH 9.6). Blocking solution contained 40mM phosphate, 0.005% (wt./vol) NaN3, 0.6% (wt./vol) NaCl, and 0.2% (wt./vol) Titriplex V (EDTA), and 1% (wt./vol) HSA (pH 8.0). Krebs Ringer bicarbonate buffer was adjusted to pH 7.4 by CO2 and supplemented with 0.1% (wt./vol) HSA. Lysis buffer contained 50 mM HEPES, 137 mM NaCl, 10 mM NaP2O7, 10mM NaF, 0.1mM MgCl2.6H20, 1 mM CaCl2.2H20, 0.1% NP-40, 10% (vol/vol) Glycerol (pH 7.4) (before use, 1 tablet of EDTA free protease inhibitor and 0.5 ml 200 mM sodium orthovanadate 14H20 was added to 50mL of lysis buffer). MAI1 and MAD1, monoclonal antibodies directed against the extracellular domain of the human IR and human IGF-IR, respectively, were obtained from Novozymes-Gropep (Adelaide, Australia). Phospho-Tyrosine Biotinylated Antibodies were obtained from R&D Systems Inc (BAM 1676; R&D Systems Europe Ltd, Abingdon, U.K.); streptavidin-labeled europium (DELFIA Eu-N1) was obtained from Perkin Elmer-life sciences (Groningen, The Netherlands). DELFIA assay reagents (assay buffer, wash buffer and enhancement solution) and a time-resolved fluorometer (Victor2 multilabel counter) were also purchased from Perkin Elmer-life sciences.

### Peptides

An aliquot of an unknown substance offered to athletes and/or their managers in 2014, was obtained and characterized by state-of-the-art analytical approaches including gel electrophoretic and mass spectrometric (top-down and bottom-up) sequencing approaches as reported elsewhere [6]. This substance turned out to have a monoisotopic molecular mass of 12264.9 Da and to be a protein, lacking the signal- and propeptide moiety, with a molecular sequence closely related to human IGF-IEc [6]: compared to human IGF-IEc the C-terminus has been modified by replacing the terminal lysine and arginine residues by a histidine residue (See Fig 1). After confirmation of its amino acid sequence, it has been denoted full-length MGF and can be considered as an analog of human IGF-IEc (Fig 1). A 1 mg lyophilisate aliquot of full-length MGF was dissolved in 1 mL of deionized water in a low-bind Eppendorf tube to obtain the stock solution, as previously described by Thevis et al.[6].

For the present study human MGF (Cat no 033–35) and its stabilized analog Goldspink-MGF (Cat no 033–34) were obtained from Phoenix Pharmaceuticals Inc., (Burlingame, USA). Noteworthy the use of the term MGF has been ambiguous in the literature in the literature, i.e. referring to IGF-1Eb/Ec mRNA, pro-IGF-1Eb, as well as the (synthetic) MGF peptide [6]. It has lately been recommended to use the name MGF solely for synthetic analogues [2]. Human recombinant IGF-I was obtained from Austral Biologicals (San Ramon, USA). Recombinant human Long-R^3^ IGF-I (Cat no 143045-27-6) was obtained from Sigma-Aldrich (Saint Louis, USA) (Fig 1). Human insulin (HI (insuman^®^) was obtained from Sanofi-Aventis (Frankfurt am Main, Germany).

### The IGF-IR, IR-A, and IR-B KIRA assays

The IGF-IR stimulating activity of full-length MGF, human MGF and Goldspink-MGF, and long-R^3^ IGF-I, respectively, were compared to IGF-I by using in-house KIRA bioassay specific for the IGF-IR. The IR-stimulating activity of full-length MGF, human MGF and Goldspink-MGF, respectively, were compared to HI by using in-house KIRA bioassays specific for the IR-A or the IR-B. The IGF-IR, IR-A and IR-B KIRA bioassays have been previously described and are based on a similar principle [8, 9, 11]. All three assays use human embryonic renal cells were stably transfected with either cDNA of the human IGF-IR gene (HEK IGF-IR) or with cDNA of the human IR-A or human IR-B gene (HEK IR-A or IR-B). Assays were performed in 48 well plates. After 48 h of culture HEK IGF-IR cells were stimulated for 15min at 37°C with an equimolar dose titration ranging from 0.0625 to 1000 moll/L of full-length MGF, human MGF and Goldspink-MGF or human recombinant IGF-I, respectively. After 48 h of culture HEK IR-A and HEK IR-B cells were stimulated for 10 min at 37°C with an equimolar dose titration ranging from 0.03125 nmol/L to 500 nmol/L of full-length MGF, human MGF and Goldspink-MGF or HI, respectively. After stimulation cells were lysed. Crude lysates were transferred to a sandwich assay. Capture antibodies MAD1 and MAI1 were used in a concentration of 5.0 μg/mL and 2.5 μg/mL, respectively. A biotinylated antiphosphotyrosine monoclonal antibody was used in a concentration of 0.2 μg/mL as detection antibody in conjunction with streptavidin-labeled europium in a concentration of 50 pmol/L. After a wash step, concentrations were read in a time-resolved fluorometer. A reference dose-titration-curve of human recombinant IGF-I (IGF-I KIRA) or human recombinant insulin (IR-A KIRA and IR-B KIRA) was implemented on all plates to ensure assay performance. All measurements were done in duplicate. The inter- and intra-assay CVs were <15% [12].

## Statistical Analysis

Data were analysed using GraphPad software (Prism 5, London, United Kingdom). The measurements of IGF-IR stimulating activity or IR-stimulating activity are presented as means ± SEM and signal-to-noise ratios. Noise was defined as signal after stimulation with vehicle and so signal-to-noise ratios were calculated by using the formula: absolute counts after stimulation with a ligand, divided by absolute vehicle counts. Differences in IGF-IR, IR-A or IR-B stimulating activity between two ligands were calculated using repeated Two Way ANOVA with Bonferroni correction. Estimation of EC50 values were calculated using a non-linear regression model for a sigmoidal dose-response curve with a variable slope. Values are presented as means with 95% CI. A two-sided p-value of <0.05 was considered statistically significant.

## Results

### A] IGF-IR stimulatory activity

#### 1. Comparison of full-length MGF vs. recombinant human IGF-I

**Fig 2:**
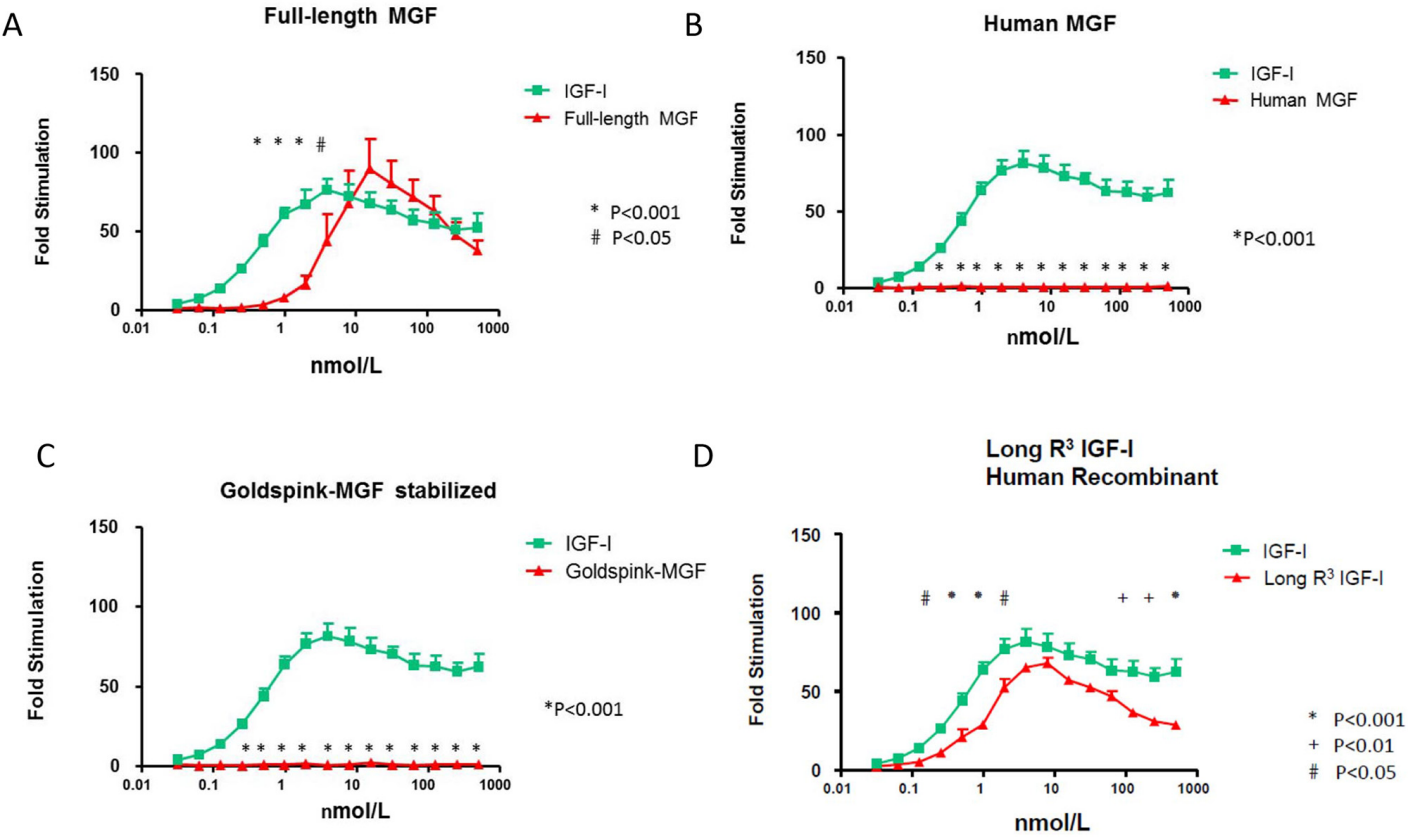
Stimulatory activity of the insulin-like growth factor-I receptor (IGF-IR). **A**: Comparison of full-length MGF (red points and line) and insulin-like growth factor-I (IGF-I) (green points and line). **B:** Comparison of Human MGF (red points and line) and insulin-like growth factor-I (IGF-I) (green points and line). **C:** Comparison of Goldspink-MGF (red points and line) and insulin-like growth factor-I (IGF-I) (green points and line). **D:** Comparison of Long R^3^ IGF (red points and line) and insulin-like growth factor-I (IGF-I) (green points and line). Dose-response curves ranged from 0.0625 to 1000 nmol/L. For bioactivity measurements means ± SEM and signal-to-noise ratios are presented. **A-C:** Each point represents the mean value ± SEM of at least three independent experiments. **D:** Each point represents the mean value ± SEM of at least two independent experiments. P = P-value when comparing overall differences in fold stimulation between two ligands.

At equimolar concentrations in the lower range full-length MGF generated significantly less IGF-IR stimulatory activity than recombinant human IGF-I (Fig 2A). The IGF-IR stimulatory activity of full-length MGF started at a concentration of 1.00 nmol/L compared to 0.0625 nmol/L for recombinant IGF-I (Fig 2A). The dose response curve of full-length MGF showed a bell-shaped curve (Fig 2A). Maximal stimulatory activity of the IGF-IR generated by full-length MGF (89-fold stimulation) was similar to that induced by recombinant human IGF-I (77-fold stimulation) and was achieved at 31.25 nmol/L for full-length MGF compared to 7.81 nmol/L for recombinant human IGF-I (Fig 2A). The EC50 values of full-length MGF and recombinant human IGF-I for the IGF-IR were 7.83 nmol/L (95% CI: 4.87–12.58) and 0.86 nmol/L (95% CI 0.69–1.07) respectively.

#### 2. Comparison Human MGF and Goldspink-MGF vs. recombinant human IGF-I

At concentrations ≥0.25 nmol/L IGF-IR stimulatory activity was significantly different between human MGF and recombinant human IGF-I (P<0.001). In contrast to recombinant human IGF-I, human MGF did not stimulate the IGF-IR at all concentrations tested (Fig 2B). Similarly, at concentrations ≥0.25 nmol/L IGF-IR stimulatory activity was significantly different between Goldspink-MGF and recombinant human IGF-I (p<0.001). In contrast to recombinant human IGF-I, Goldspink-MGF did not stimulate the IGF-IR at any concentrations tested (Fig 2C).

#### 3. Comparison of Recombinant human Long- R^3^ IGF-I vs. recombinant human IGF-I

The dose response curve of recombinant human long R^3^ IGF showed a bell-shaped curve (Fig 2D). The IGF-IR stimulatory activity of recombinant human Long-R^3^ IGF-I started at a concentration of 0.125 nmol/L compared to 0.0625 nmol/L for recombinant IGF-I. At equimolar concentrations in the lower range and the higher range human long R^3^ IGF-I generated significantly less IGF-IR stimulatory activity than recombinant human IGF-I (Fig 2D). However, maximal stimulatory activity of the IGF-IR generated by Long-R^3^ IGF (68-fold) was similar to that induced by recombinant human IGF-I (82-fold stimulation) (Fig 2D). Maximal IGF-IR stimulation was achieved at 3.91 nmol/L for recombinant human IGF-I and at 7.81 nmol/L for recombinant human Long- R^3^ IGF-I (Fig 2D). The EC50 value of recombinant human recombinant IGF- and Long-R^3^ IGF-I for the IGF-IR were 0.94 nmol/L (95% CI 0.74–1.19) and 1.92 nmol/L (95% CI 1.52–2.43), respectively.

### B] IR-A stimulatory activity

#### 1. Comparison of full-length MGF vs. recombinant human insulin

**Fig 3:**
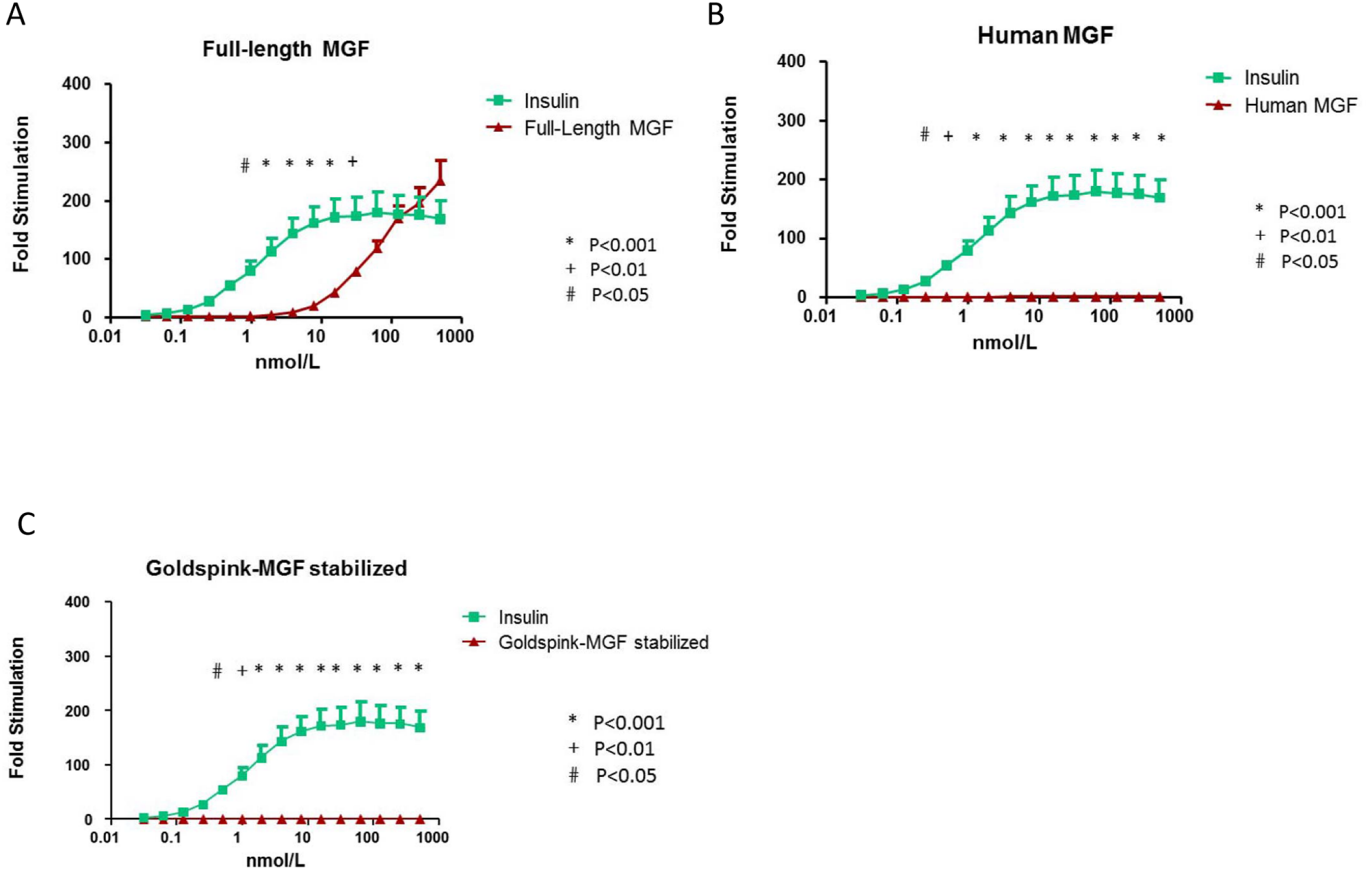
Stimulatory activity of the insulin receptor-A (IR-A). **A**: Comparison of full-length MGF (red points and line) and insulin (green points and line). **B:** Comparison of Human MGF (red points and line) and insulin) (green points and line). **C:** Comparison of Goldspink-MGF (red points and line) and insulin (green points and line). Dose-response curves ranged from 0.03125 nmol/L to 500 nmol/L. For bioactivity measurements means ± SEM and signal-to-noise ratios are presented. **A-C**: Each point represents the mean value ± SEM of at least three independent experiments. P = P-value when comparing overall differences in fold stimulation between two ligands.

The IR-A stimulatory activity of full-length MGF started at a concentration of 4.00 nmol/L compared to 0.0625 nmol/L for recombinant human insulin (Fig 3A). In the range between 0.98 and 31.25 nmol/L IR-A stimulatory activity for full-length MGF was significantly lower than that of recombinant human insulin. However, maximal stimulatory activity of the IR-A generated by ‘full-length MGF’ (235-fold stimulation) was similar to that generated by recombinant human insulin (193-fold stimulation) and was achieved at 500.0 nmol/L for full-length MGF and at 62.50 nmol/L for recombinant human insulin (Fig 3A). The EC50 values of full-length MGF and recombinant human insulin for the IR-A receptor were 73.11 nmol/L (42.87–124.69) and 1.13 nmol/L (95% CI 0.69–1.84), respectively.

#### 2. Comparison of Human MGF and Goldspink-MGF vs. recombinant human insulin

At concentrations ≥0.50 nmol/L IR-A stimulatory activity was statistically different between human MGF and recombinant human insulin (Fig 3B). In contrast to recombinant human insulin, human MGF did not stimulate the IR-A at all concentrations tested (Fig 3B). At concentrations ≥0.50 nmol/L IR-A stimulatory activity was statistically different between Goldspink-MGF and recombinant human insulin (P<0.001) (Fig 3C). In contrast to recombinant human insulin, Goldspink-MGF did not stimulate the IR-A at all concentrations tested (Fig 3C).

### C] IR-B stimulatory activity

#### 1. Comparison of full-length MGF vs. recombinant human insulin

**Fig 4:**
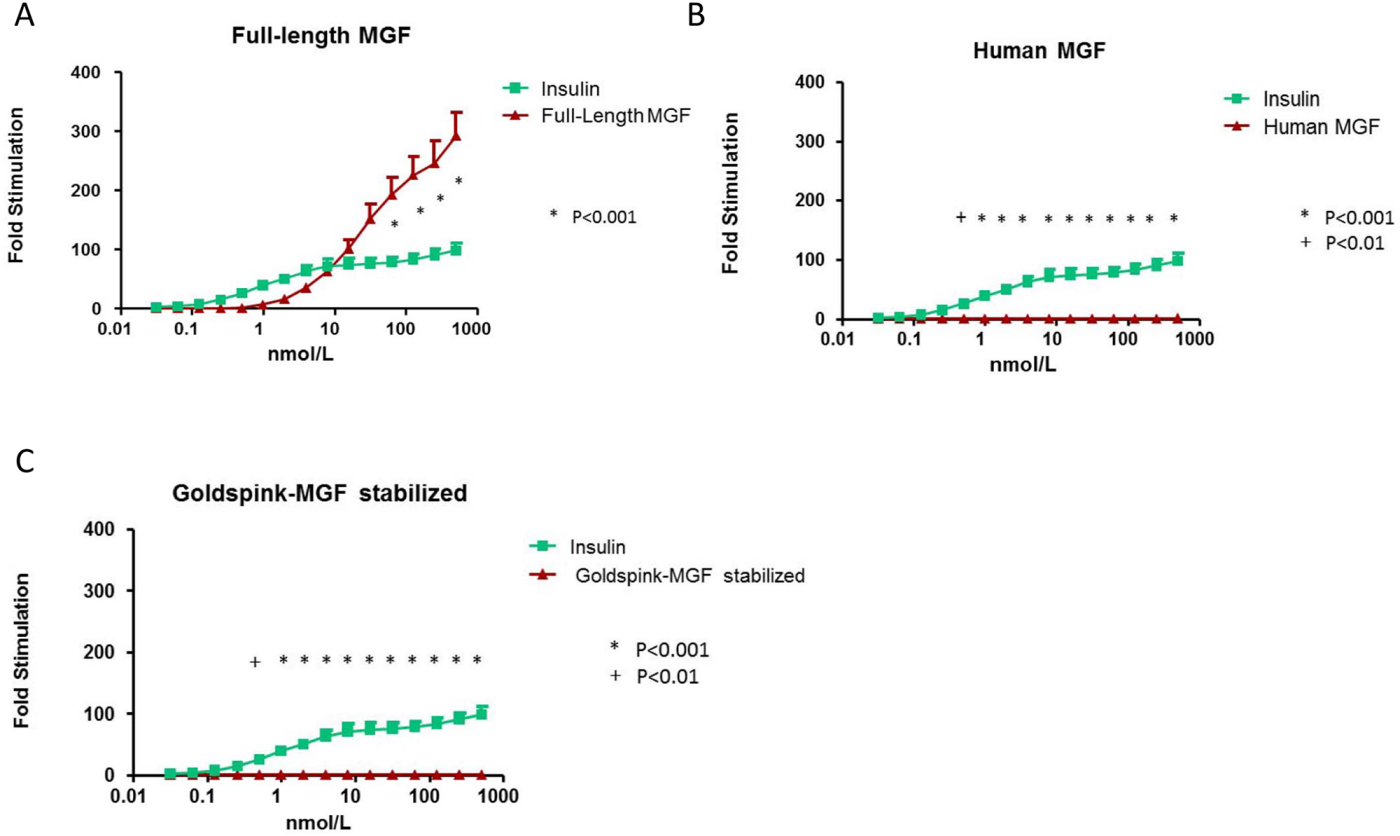
Stimulatory activity of the insulin receptor-B (IR-B). **A:** Comparison of full-length MGF (red points and line) and insulin (green points and line). **B:** Comparison of Human MGF (red points and line) and insulin) (green points and line). **C:** Comparison of Goldspink-MGF (red points and line) and insulin (green points and line). Dose-response curves ranged from 0.03125 nmol/L to 500 nmol/L. For bioactivity measurements means ± SEM and signal-to-noise ratios are presented. **A-C:** Each point represents the mean value ± SEM of at least three independent experiments. P = P-value when comparing overall differences in fold stimulation between two ligands.

The IR-B stimulatory activity of full-length MGF started at a concentration of 0.98 nmol/L compared to 0.0625 nmol/L for recombinant human insulin (Fig 4A). Full-length MGF at equimolar concentrations in the lower concentration range generated less IR-B stimulatory activity than recombinant human insulin, but at equimolar concentrations in the upper concentration range had significantly higher IR-B stimulatory activity than recombinant human insulin (Fig 4A). Maximal IR-B stimulatory activity of full-length MGF was considerably higher (292-fold) than that induced by recombinant human insulin (98-fold) and this was achieved at the maximum dose tested of 500.0 nmol/L for both full-length MGF and recombinant human insulin (Fig 4A). The EC50 values of full-length MGF and recombinant human insulin for the IR-B receptor were 35.91 nmol/L (95% 17.52–70.33) and 1.28 nmol/L (95% CI 0.64–2.57), respectively.

#### 2. Comparison of Human MGF and Goldspink-MGF vs. recombinant human insulin

At concentrations ≥0.50 nmol/L IR-B stimulatory activity was statistically different between human MGF and recombinant human insulin (P<0.001) (Fig 4B). In contrast to recombinant human insulin, human MGF did not stimulate the IR-B at all concentrations tested (Fig 4B). Similarly, at concentrations ≥0.50 nmol/L IR-B stimulatory activity was statistically different between Goldspink-MGF and recombinant human insulin (P<0.001) (Fig 4C). In contrast to recombinant human insulin, Goldspink- MGF did not stimulate the IR-B at all concentrations tested (Fig 4C).

## Discussion

When we tested in vitro potency with our in house IGF-IR KIRA assay, full -length MGF at equimolar concentrations in the lower ranges generated less IGF-IR stimulatory activity than recombinant human IGF-I, suggesting that the IGF-IR is less sensitive to full -length MGF than to recombinant human IGF-I. However, despite a higher EC50 concentration, at high equimolar concentrations full-length MGF showed a similar maximal potency to activate the IGF-IR than IGF-I (Fig 2A).

The IGF-I KIRA assay measures a physiologically meaningful parameter: quantification of the phosphorylated tyrosine residues within the ß-subunits of the IGF-IR. Phosphorylation of the tyrosine residues of the ß-subunits of the IGF-IR initiates the majority, if not all of the biological effects mediated by the IGF-IR. On the other hand, the IGF-IR KIRA assay provides no direct information about intracellular post-receptor effects. For example, we do not know whether activation of the IGF-I receptor by full- length MGF results (like human IGF-I) in more mitogenic as opposed to metabolic activity of the IGF-I receptor.

The molecular structure of full-length MGF shows considerable homology with IGF-1Ec, which is one of three known IGF-I isoforms, that are naturally occurring in humans [6] (see also Fig 1). The intact molecular weight of full-length MGF is 147, 2 Da lower than that of human IGF-1Ec. This discrepancy in molecular weight is due to a slight structural difference between both substances in molecular structure. Full- length MGF is one amino acid shorter and contains a histidine residue at the carboxy-terminus instead of arginine and lysine [6]. Full-length MGF can thus be considered as a pro-IGF-I with slight modifications. Our finding that full-length MGF directly stimulates the IGF-IR, challenges the current concept that the E-peptide of pro-IGF-I has to be cleaved off from the IGF-I molecule to become biologically active [13, 14].

The residence time in vivo of a given glycoprotein is often dependent on its glycosylation status [15]. On the other hand, a lack of glycosylation may also reduce the ability of a ligand to activate a receptor [1]. Thus, the glycosylation state of MGF may be an important factor and it has been suggested that glycosylated MGF directly associates with the extracellular matrix (ECM), thereby serving as an alternate additional reservoir of this growth factor: as such glycosylation may attribute to a delayed clearance, while cleavage of the entire E-peptide by extracellular proteases could release active mature IGF-I for receptor binding when needed [1]. However, the full-length MGF molecule we studied appeared to be a pure peptide structure which is not glycosylated [6]. It also does not show any additional signals or indications for glycan residues [6]. Our results support previous observations that IGF-IR stimulatory activity of mature IGF-I and non-glycosylated MGF are similar [1].

The stimulatory effects on the IGF-IR in the KIRA assay occurred at relatively high molar concentrations of full-length MGF. However, at present no information is available about the in vivo concentrations of MGF at the IGF-I receptor site. In the physiological states in the human body MGF probably does not enter the bloodstream in any significant quantity, but remains in the muscular compartment [4]. It has been suggested that the E-peptide may prevent release of MGF into the bloodstream by tethering MGF to the site of synthesis and/or administration [16]. This would help to increase the local actions of MGF [4]. In this context, the recently reported findings for polyethylene glycol (PEG)ylated IGF-I are very interesting: it has been demonstrated that a single intramuscular injection of PEG-IGF-I was more efficacious than systemic administration of PEG-IGF-I or a single intramuscular injection of recombinant human IGF-I for hastening early muscle fiber regeneration and improving muscle function after injury [17]. This latter effect was attributed to a delayed clearance of PEG-IGF-I after intramuscular injection and suggests that molecular modifications near the C terminus of the IGF-I molecule are very important to increase the actions of IGF-I at the tissue level [1, 17]. Whether full-length MGF in this respect resembles PEG-IGF-I is not clear at present and should be studied.

It would be very interesting to determine whether full-length MGF and IGF-I differ in their kinetics of IGF-IR activation. However, due to a limited availability of full-length MGF, we could not directly assess IGF-IR activation kinetics of full-length MGF to compare it with IGF-I.

It is well established that Human MGF and Goldspink-MGF are synthetic IGF-I E peptide analogues corresponding to the last 24 amino acids of the E domain of the IGF-IEc isoform [6]. It has been suggested that these E peptides may function as independent growth factors [18]. In our hands, human MGF and Goldspink-MGF in the IGF-IR KIRA assay induced no stimulatory activity of the IGF-IR. In line with our findings, it has been found that the E-peptides do not directly induce IGF-IR phosphorylation [19, 20]. Although in a rodent model, exposure to E- peptides has been shown to increase myoblast proliferation and migration, many of these effects were apparent even when IGF-IR was blocked via a neutralizing antibody, indicating that these E-peptide actions were independent of IGF-IR signaling [19].

Long-R^3^ IGF-I is derived from native IGF-I by replacing the third amino acid in the mature peptide, glutamine, with arginine [21]. This substitution gives Long-R^3^ IGF-I a >100-fold reduced affinity for IGFBPs without compromising IGF-IR affinity [22]. At both low and high equimolar concentrations, Long -R^3^ IGF-I was less potent to activate of the IGF-IR than recombinant human IGF-I. However, maximal stimulatory activity of the IGF-IR generated by Long-R^3^ IGF (68-fold) was similar to that induced by recombinant human IGF-I and the dose-response curve of Long-R^3^ IGF-I was bell-shaped [21]. Despite our findings, Long-R^3^ IGF-I may still act in vivo as a more potent growth factor than native IGF-I, due to its considerably reduced affinities for the IGFBPs in plasma [23, 24]. It has been reported that Long-R^3^ IGF-I demonstrated an increased bioavailability and potency at the target tissues due to its faster clearance from blood into the target tissues in comparison to native IGF-I [23, 24].

The structures of the IRs and the IGF-IR resemble each other to such an extent that insulin and IGFs can interact with the respective other receptor, although with quite different affinities and actions [25]. This may be also true for IGF-I isoforms. Since full-length MGF shows considerable structural similarities with mature IGF-I, we also investigated whether full-length MGF may stimulate the IRs and observed that full -length MGF also stimulated the IR-A and IR-B. The IR-A and the IR-B stimulatory activity started at higher concentrations of full-length MGF compared to those inducing IGF-IR stimulatory activity. In addition, at equimolar concentrations in the lower range, sensitivity of the IRs to stimulation with full-length MGF was less compared to human insulin. However, it is important to note that at high equimolar concentrations similar maximal IR-A stimulating effects were found for full -length MGF and human insulin, however, maximal stimulatory activity achieved for the IR-B was stronger after full- length MGF than by equimolar doses of human insulin. In contrast to the above described effects for the IGF-IR, the dose-response curves of full-length MGF for both IRs showed no peak/plateau at the concentrations tested and the stimulation of the IRs progressively increased with higher concentrations of full-length MGF. In addition, for both IRs the maximal stimulatory activity induced by full-length MGF apparently was not reached at the highest concentration of full length MGF tested (Figs 3A and 4A).

Although a limitation of this study may be that affinities of the IGF-IR and the IRs for full-length MGF were not measured, biological responses do not parallel ligand binding and, therefore, are not exclusively determined by the affinity of a particular receptor for that ligand [26]. In addition, the magnitude of a cellular response to a ligand is determined by summation of all the signals generated by each of the occupied receptors, and therefore is related to number of receptors that are activated rather than the fraction of the total receptor pool that is bound by the ligand [26]. With the KIRA assays the total amount of autophosphorylation of tyrosine residues of a particular receptor (IGF-IR, IR-A and IR-B) is measured which initiates most if not all of the biological effects of these receptors. Therefore, the KIRA assays produce physiologically more relevant information than the assessment of the affinities of the IGF-IR and the IRs

As previously discussed for the IGF-IR, both IR KIRA assays do not produce information about intracellular post-receptor events. At present we do not know which metabolic and mitogenic responses are induced by activation of the IRs by full-length MGF. While it is apparent from our study that full-length MGF induces a phosphorylation pattern of the IR-B, which is significantly different from that of insulin, the potential consequences of this different phosphorylation pattern for the human body in vivo are not yet obvious.

At present it is not clear to what extent our in vitro findings about full length MGF can be translated into biological responses of cells in vivo and whether this substance may improve skeletal muscle function and/or hypertrophy. New studies in animals should be initiated to gather in vivo information about efficacy, toxicity and pharmacokinetics of full-length MGF.

In conclusion, full-length MGF in vitro at equimolar concentrations in the lower range demonstrated lower activation of the IGF-IR than IGF-I. However, despite a higher EC50 concentration, at equimolar concentrations in the higher ranges maximal IGF-IR stimulatory activity achieved by full-length MGF was similar (90-fold) to that after recombinant human IGF-I (82-fold). No IGF-IR stimulatory activity was induced by human MGF and Goldspink-MGF, respectively.

At high equimolar concentrations similar maximal levels of IR-A stimulating effects were observed for full-length MGF and human insulin, but maximal stimulatory activity achieved for the IR-B was stronger after full- length MGF than after adding equimolar doses of human insulin. Both the IRs showed no clear peak/plateau in stimulatory activity and the stimulatory activity of the IRs progressively increased with higher concentrations of full-length MGF.

Further research is needed to better understand the actions of full-length MGF in vivo and to place our in vitro findings into the context of the actions of full-length MGF in vivo.
